# Asia-Pacific consensus on the use of artificial intelligence in colorectal cancer screening and surveillance

**DOI:** 10.1016/j.igie.2025.04.001

**Published:** 2025-04-16

**Authors:** Frederick H. Koh, James W. Li, Sunny H. Wong, Jonathan Lee, Sneha John, Vui Heng Chong, Kai-chun Wu, Rashid Lui, Simon S.M. Ng, Thomas Y.T. Lam, Louis H.S. Lau, Govind K. Makharia, Murdani Abdullah, Hasan Maulahela, Nozomu Kobayashi, Masau Sekiguchi, Jeong-Sik Byeon, Hyun-soo Kim, Yeong Yeh Lee, Han-Mo Chiu, I-Chen Wu, Somchai Leelakusolvong, Prateek Sharma, David Lieberman, Joseph J.Y. Sung

**Affiliations:** 1 Sengkang General Hospital, Singapore; 2 Changi General Hospital, Singapore; 3 Lee Kong Chian School of Medicine, Nanyang Technological University, Singapore; 4 Department of Gastroenterology and Hepatology, Tan Tock Seng Hospital, Singapore; 5 Division of Gastroenterology and Hepatology, National University Health System, Singapore; 6 Department of Medicine, Yong Loo Lin School of Medicine, National University of Singapore, Singapore; 7 Institute for Health Innovation & Technology (iHealthtech), National University of Singapore, Singapore; 8 Gold Coast University Hospital, Southport, Australia; 9 Raja Isteri Pengiran Anak Saleha Hospital, Bandar Seri Begawan, Brunei Darussalam; 10 The Fourth Military Medical University, Xian, China; 11 Prince of Wales Hospital, Hospital Authority, Hong Kong, China; 12 The Chinese University of Hong Kong, Hong Kong, China; 13 Nethersole School of Nursing, The Chinese University of Hong Kong, Hong Kong, China; 14 All India Institute of Medical Sciences, New Delhi, India; 15 Division of Gastroenterology, Department of Internal Medicine, Faculty of Medicine, Universitas Indonesia, Jawa Barat, Indonesia; 16 Cipto Mangunkusumo National General Hospital, Jakarta, Indonesia; 17 National Cancer Center Hospital, Tokyo, Japan; 18 University of Ulsan College of Medicine, Seoul, South Korea; 19 Yonsei University, Seoul, South Korea; 20 School of Medical Sciences, Universiti Sains Malaysia, Kota Bharu, Malaysia; 21 National Taiwan University, Taipei, Taiwan; 22 Kaohsiung Medical University Hospital, Kaohsiung Medical University, Kaohsiung, Taiwan; 23 Siriraj and Siriraj Piyamaharajkarun Hospital, Mahidol University, Bangkok, Thailand; 24 VA Medical Center and the University of Kansas School of Medicine, Kansas City, Kansas, USA; 25 Oregon Health & Science University, Portland, Oregon, USA

## Abstract

**Background and Aims:**

Artificial intelligence (AI)-assisted colonoscopy has been widely investigated for colorectal adenoma and cancer detection and characterization. However, clinical guidance on its use in colorectal cancer (CRC) screening and surveillance is lacking. In this study, we developed consensus guiding when and how to use AI-assisted colonoscopy in the daily practice of screening and surveillance of colorectal neoplasia.

**Methods:**

Experts from 12 Asian-Pacific countries and regions together with 4 international experts developed a set of consensus statements based on existing clinical evidence using the modified Delphi process.

**Results:**

Based on existing evidence, computer-assisted detection should be evaluated for use if available and deemed cost-effective as an adjunct to conventional colonoscopy for CRC screening. Computer-assisted diagnosis may be used for characterization of polyps but is not yet considered sufficient to determine if a polyp is neoplastic and needs to be removed. Computer-assisted quality assurance is generally welcome to improve the quality of colonoscopy. Early engagement of patients and nurses and training of endoscopists to use AI-assisted colonoscopy are crucial for the successful implementation.

**Conclusions:**

More validation studies on AI-assisted colonoscopy need to be done while AI technologies continue to improve.

Colorectal cancer (CRC) is the third most common cancer and the second leading cause of cancer-related death worldwide. The Asia-Pacific region contributes the largest burden of cases of CRC (51.8%) and CRC mortality (52.4%).^1^

Artificial intelligence (AI) is becoming more widely used and accepted in medical care. The American Gastroenterological Association’s Center for Gastrointestinal Innovation and Technology released a White Paper in 2022 highlighting the utility of AI in CRC screening through colonoscopy.^2^ There has yet to be any consensus guiding the application of AI in colonoscopy for CRC screening, despite the region contributing the most CRC cases annually worldwide.

In view of the current developments around AI in colonoscopy, the Asia-Pacific Working Group on Colorectal Cancer Screening decided to convene a consensus meeting in 2023 to summarize the latest evidence of the use of AI in CRC screening. We then used this evidence to support recommendation statements and guide endoscopists in the Asia-Pacific region on the implementation of this technology in CRC screening.

## Methods

The consensus was conducted as a hybrid meeting in conjunction with the Asia-Pacific Digestive Week 2023 in Bangkok. The process was carefully designed to strictly comply with a modified Delphi process.

### Membership of the consensus panel

Members of the consensus group were selected based on the following criteria: demonstrated knowledge and/or expertise in CRC screening by publication and research or participation in development of national or regional guidelines, geographic representation of various Asia-Pacific countries and regions, participation in the Asia-Pacific Working Group on Colorectal Cancer Screening research projects and/or previous Asia-Pacific consensus recommendation process in the past, and leaders in the field of AI who had experience and/or published on the topic of AI-assisted colonoscopy. International experts who have played key roles in drafting other regional, national, and/or international guidelines were invited to contribute to the drafting, discussion, and voting for these recommendations. Membership was divided into 4 roles: convenors (J.J.Y.S., F.H.K., J.W.L., J.L., and S.H.W.) were responsible for drafting statements, coordinating the process of discussion, and voting on the statements; international advisors (P.S. and D.L.) helped to draft the first statements, moderate discussion during the meeting, and revise the statements accordingly; panelists were country or region representatives who participated in the discussions and voting of the statements during the virtual conference (S.J., V.H.C., K.C.W., R.L., S.S.M.N., T.Y.T.L., L.L., G.K.M., M.A., H.M., N.K., M.S., J.S.B., H.S.K., Y.Y.L., H.M.C., I.C.W., and S.L.); and facilitators (F.H.K., J.W.L., J.L., and S.H.W.) were responsible for performing the literature search, collating the discussion points, and revising the statements.

### Provisional statements

The consensus was grouped under 4 topics, namely, the use of computer-assisted detection (CADe) programs in colonoscopy for CRC screening, use of computer-assisted diagnosis (CADx) programs for CRC screening, use of computer-assisted quality assurance (CAQ) programs for CRC screening, and implementation and education of computer-assisted colonoscopy into routine clinical practice.

### Literature search

A comprehensive literature search was conducted by the facilitators. Relevant articles published in the English language from 2018 to October 2023, using EMBASE, Ovid Medline, PubMed Medline, Cochrane Library, and Google Scholar, were included. National and international guidelines on the use of AI-assisted colonoscopy were also solicited. Both the full articles and a summary of the literature search were provided to the panel before the voting process for their reference.

### Voting process

The voting process was adopted from our previous Asia-Pacific consensus.^3^ A Likert scale of 1 to 5 was adopted (Table 1). Consensus was achieved when >80% of the votes indicated “accept completely” or “accept with some reservation.” A statement was refuted when >80% of the votes indicated “reject completely” or “reject with some reservation.” Statements for which a consensus could not be reached were discussed and modified at the face-to-face meeting. A second round of anonymized electronic voting was then conducted. If consensus was still not reached, the statement was refuted. Each statement was then graded to indicate the level of evidence available and the strength of recommendation (Table 1).

**Table 1:** Level of agreement, quality of evidence, and classification of recommendations

Category and grade	Description
Likert scale level of agreement with statement	
A	Accept completely
B	Accept with some reservation
C	Accept with major reservation
D	Reject with some reservation
E	Reject completely
Quality of evidence	
I	Evidence obtained from at least 1 randomized controlled trial
II-1	Evidence obtained from well-designed control trials without randomization
II-2	Evidence obtained from well-designed cohort or case-control study
II-3	Evidence obtained from comparison between time or places with or without intervention
III	Opinion of respected authorities, based on clinical experience and expert committees
NA	Not applicable
Classification of recommendations	
A	There is good evidence to support the statement
B	There is fair evidence to support the statement
C	There is poor evidence to support the statement but recommendation made on other grounds
D	There is fair evidence to refute the statement
E	There is good evidence to refute the statement

## Results

A 1-day hybrid consensus conference was held on December 9, 2023. Representatives from 12 Asia-Pacific countries and regions participated in the meeting, including Australia, Brunei, China, Hong Kong, India, Indonesia, Japan, Korea, Malaysia, Singapore, Taiwan, and Thailand. Four international experts from Italy, Norway, and the United States also participated. Twenty-three members took part in anonymized digital voting.

### Computer-aided detection

Statement 1. CADe has been shown to improve adenoma detection.

Level of agreement: A = 74.1%, B = 18.5%, C = 3.7%, D = 0%, E = 3.7%

Quality of evidence: I

Classification of recommendation: A

The advent of CADe has provided an adjunct for colorectal polyp detection. Randomized controlled trials (RCTs) have been performed using various CADe systems, a large proportion of which (12/21, 57%) were performed in the Asia-Pacific region (Table 2).^4^, ^5^, ^6^, ^7^, ^8^, ^9^, ^10^, ^11^, ^12^, ^13^, ^14^, ^15^, ^16^ A recent meta-analysis summarized the outcomes of these trials.^17^ There was a 24% improvement in adenoma detection rate (ADR) (95% CI, 1.16-1.33) using CADe. Despite the heterogeneity of the included studies and wide range in improvement in ADR (12%-70%), the pooled results suggest that the application of CADe is generalizable across different practices, taking into account variation in endoscopist experience and type of CADe system available. In addition, CADe has been shown to be most useful in endoscopists who have low ADRs in improving the quality of colonoscopy. Subsequent RCTs, published after the inclusion window of the above meta-analysis, confirmed the improvement of ADRs with CADe.^4^, ^5^, ^6^ RCTs that specifically look at screening colonoscopies also showed an improvement in ADRs with CADe without prolonging procedural time. The few studies that did not demonstrate this were assessed to have a methodologic bias.^7^

**Table 2:** Summary of CADe-related randomized controlled trials in the last 5 years

Author	Year of publication	Country/region involved	Sample size (CADe vs standard colonoscopy)	CADe program used	Adenoma detection rate outcomes
Wang et al^8^	2019	China	522 vs 536	EndoScreener	29.1% vs 20.3%
Liu et al^9^	2020	China	397 vs 393	EndoScreener	29.01% vs 20.91%
Gong et al^10^	2020	China	355 vs 349	ENDOANGEL	16% vs 8%
Shaukat et al^11^	2022	USA	682 vs 677	SKOUT	47.8% vs 43.9%
Glissen Brown et al^12^	2022	USA	116 vs 116	EndoScreener	50.44% vs 43.64%
Karsenti et al^4^	2023	France	1013 vs 1026	GI Genius	37.5% vs 33.7%
Wang et al^6^	2023	China	636 vs 625	EndoScreener	25.8% vs 24.0%
Nakashima et al^5^	2023	Japan	207 vs 208	CADEYE Fujifilm	59.4% vs 47.6%
Gimeno-Garcia et al^13^	2023	Spain	185 vs 185	ENDO-Aid	55.1% vs 43.8%
Lui et al^14^	2023	Hong Kong, Vietnam, Singapore	108 vs 108	NISI(HK)	44.7% vs 34.6%
Wei et al^7^	2023	USA	387 vs 382	GI Genius	35.9% vs 37.2%
Xu et al^15^	2023	China	1519 vs 1540	EagleEye	39.9% vs 32.4%
Lau et al^16^	2024	Hong Kong	386 vs 380	ENDO-Aid	57.5% vs 44.5%

*CADe,* Computer-assisted detection.

Statement 2. CADe should be used if available as a preferred choice over conventional colonoscopy for CRC screening.

Level of agreement: A = 37.0%, B = 44.4%, C = 14.8%, D = 3.7%, E = 0%

Quality of evidence: II-2

Classification of recommendation: C

Although CADe has been demonstrated in RCTs to improve ADR, the recommendation on whether it should be the “preferred” choice in CRC screening has to consider other issues such as efficiency and cost-effectiveness. Studies have shown that the use of CADe in routine colonoscopy did not significantly increase procedural time despite its increased ADR or adenoma per colonoscopy (APC).^18^ The cost of CADe will have a direct effect on the adoption of this technology, especially in resource-limited countries and regions. So far, health economic studies suggest that CADe programs are cost-effective in reducing missed adenomas or colon cancer.^19^^,^^20^ However, cost-effectiveness analyses are highly dependent on the intended outcome and may not be entirely comprehensive or applicable in every clinical situation.

There is significant disparity in the cost of endoscopy between economically developed and developing countries in the Asia-Pacific region. Healthcare reimbursement and access to funding also varies among the healthcare systems of individual countries, which may be predominantly government funded or self-funded. Recommendation on the use of AI-assisted colonoscopy should consider healthcare equity issues in the region. More region-specific health economic analyses should be performed to inform healthcare authorities.

Because data are lacking that show the impact of CADe on postcolonoscopy CRC, the consensus group voted for a marginal support (81.4%) and stated that further studies with a longer follow-up duration are required. Establishment of clinical efficacy in reducing CRC incidence and mortality beyond ADR improvement is mandatory. In regions where CADe is both available and cost-effective, one should consider the use of CADe in colonoscopy to leverage the potential for improving polyp detection afforded by CADe.

The consensus group is cognizant about the potential negative impacts and drawbacks of CADe such as false-positive findings and the risk of over-reliance on AI technology. Other knowledge gaps need to be filled such as finding of sessile serrated lesions (SSLs; see below) or detection of cancer in inflammatory bowel diseases.

Statement 3. In using CADe for CRC screening, APC should be considered as an additional indicator of quality.

Level of agreement: A = 33.3%, B = 66.7%, C = 0%, D = 0%, E = 0%

Quality of evidence: II-1

Classification of recommendation: C

The use of ADR as a performance index for colonoscopies has been the criterion standard to predict CRC. However, the use of ADR alone may not truly reflect the performance of CRC screening programs. Adenoma miss rate (AMR) reflects the negative side of poor-quality colonoscopy. Typically, AMR can only be ascertained in tandem studies where colonoscopy both with and without CADe is performed on the same patient. Thus, reporting AMR may not be possible in routine practice.

The number of APCs is a useful parameter that may help in estimating the risk of CRC.^17^ CADe programs have been suggested to not only help endoscopists find more patients with adenoma but also to increase the number of adenomas detected in each patient. A recent study published by Anderson et al^21^ reported that with increased APC, postcolonoscopy CRC was reduced. Reporting the APC may be a more appropriate surrogate for AMR in nontandem studies. As such, to truly appreciate the effectiveness of CADe, future publications around its use should consider reporting both ADR and APC.

Although consensus was reached for this statement, it was noted that only one-third of members support this notion without reservation. More studies reporting both ADR and APC should be conducted to fully appreciate the utility and effectiveness of CADe in CRC screening.

### Computer-aided diagnosis

Statement 4. CADx can be used for characterization of polyps but is not yet ready to determine the necessity of polypectomy.

Level of agreement: A = 70.4%, B = 25.9%, C = 3.7%, D = 0%, E = 0%

Quality of evidence: II-2

Classification of recommendation: B

Optical diagnosis of the nature of colorectal polyps during colonoscopy enables clinicians to decide on the management of those polyps during the procedure. The potential capability of CADx in characterizing polyp histology without the need for biopsy sampling and histology have sparked debate on the strategies of “diagnose-and-leave” and “resect-and-discard.” The Preservation and Incorporation of Valuable Endoscopic Innovations (PIVI) established by the American Society for Gastrointestinal Endoscopy sets thresholds when these policies can be acceptable.^22^ The first PIVI threshold states that for a “diagnose-and-leave” strategy in the management of rectosigmoid hyperplastic polyps ≤5 mm, CADx should have a negative predictive value (NPV) for adenomatous histology of ≥90%.^22^ The second PIVI threshold for “resect-and-discard” mandates that the agreement in recommending postpolypectomy surveillance intervals between predicted histology determined by CADx for colorectal polyps ≤5 mm, when combined with the histology of polyps >5 mm, is ≥90% compared with that obtained from the histologic assessment of all resected polyps.^23^

Commercially available CADx systems such as CAD-EYE (Fujifilm, Minato-ku, Tokyo, Japan), GI-GENIUS (Medtronic, Minneapolis, Minn, USA), and EndoBRAIN (Olympus, Hachioji-shi, Tokyo, Japan), which have been evaluated in clinical studies, are currently only able to generate a binary classification of either neoplastic or non-neoplastic (Table 3). Current trials using these CADx systems are quite heterogenous in their study design (clinical trial environment vs real-world practice, single-center vs multicenter) and are mostly open-label single-arm studies. Studies in Japan and Italy suggested that CADx not only improved the characterization of polyps but was also able to satisfy PIVI thresholds for leaving diminutive rectosigmoid polyps in situ, sparing the need for unnecessary polypectomy.^20^^,^^24^ In a multicenter study, CADx was also demonstrated to increase the proportion of optical diagnoses made with high confidence from 74.2% to 92.6%.^25^ However, subsequent clinical studies conducted in multicenter settings have demonstrated mixed and even conflicting results with regard to CADx predictions of polyp histology and PIVI endpoints. Although the NPV of AI-assisted optical diagnosis for adenomatous histology in diminutive rectosigmoid polyps was 91% in an Italian multicenter study, the NPV for AI-alone diagnosis fell short of this threshold at 86.7%. A more recent Singapore study also showed similar findings, that is, CADx having an NPV of 80.6% for the “leave in situ” PIVI threshold.^26^ A third multicenter study reported that the NPV for diminutive rectosigmoid polyps by CADx was only 66.7%, which fell short of the PIVI threshold and was inferior to the performance of endoscopists with a NPV of 78.6% (95% confidence interval, 63.4-93.8).^27^ A prospective study conducted across 6 centers in the United States also showed that CADx did not increase the sensitivity of optical diagnosis in general endoscopists with a suboptimal specificity.^28^ Other studies show variable results especially on NPV of diagnosing neoplasms.^29^, ^30^, ^31^, ^32^ A systematic review and meta-analysis of 10 studies demonstrated no difference in the proportion of polyps predicted to be non-neoplastic that could avoid polypectomy with a CADx-assisted strategy compared with a CADx-unassisted strategy (58.4% vs 55.4%).^33^ Given the heterogeneity in study design and data quality, the consensus group believed that it is premature to recommend CADx as a guidance to polypectomy and the need of histologic confirmation.

**Table 3:** Studies testing the use of CADx to characterize colorectal polyps including DRSPs

Study	Study design	Sample size	Results
Hassan et al, 2022^20^	Single-center, single-arm, prospective	162	NPV for DRSPs 97.6% (95% CI, 94.1-99.1) for CADx
Mori et al, 2018^24^	Single-group, open-label, prospective	325	NPV for diminutive rectosigmoid adenomas 93.7%-96.4% for CADx with stained mode
Barua et al, 2022^25^	Multicenter, open-label, prospective	518	Sensitivity for neoplastic polyps 90.4% (95% CI, 86.8-93.1) with CADx compared with 88.4% (95% CI, 84.3-91.5) with standard visual inspection
Li et al, 2023^26^	Multicenter, real-world, prospective clinical validation study	320	CADx overall accuracy 71.6% (95% CI, 68.0-75.0) compared with 75.2% (95% CI, 71.7-78.4) for endoscopists; accuracy increased to 78.1% when there was concordance between CADx and endoscopist predictions of polyp histology
Houwen et al, 2023^27^	Multicenter, prospective	194	CADx accuracy 79.4% (95% CI, 75.2-83.2) compared with 83.0% (95% CI, 79.1-86.4) for endoscopists
Rex et al, 2024^28^	Multicenter, single-group, prospective	1252	CADx-assisted sensitivity 90.8% (95% CI, 89.3-92.3) compared with 90.7% (95% CI, 89.2-92.2) for endoscopists
Minegishi et al, 2022^29^	Single-center, single-arm, open-label, prospective	181	NPV for rectosigmoid neoplasms (adenomas and sessile serrated lesions) with CADx 94.1% (95% CI, 83.8-98.8) when used with high confidence
Rondonotti et al, 2023^30^	Multicenter, prospective, cohort study	389	NPV of artificial intelligence–assisted optical diagnosis for DRSPs 91.0% (95% CI, 87.1-93.9)
Hassan et al, 2023^31^	Prospective, head-to-head comparison trial of 2 commercially available CADx systems (CADx-A and CADx-B)	176	NPV for DRSPs 97.0% (95% CI, 95.0-99.0) and 97.7% (95% CI, 95.9-99.5) for CADx-A and CADx-B, respectively
Baumer et al, 2023^32^	Single-center, nonrandomized, prospective	156	CADx accuracy 84.35% (95% CI, 79.38-88.53) compared with 83.10% (95% CI, 72.34-90.95) for less-experienced endoscopists and 83.77% (95% CI, 77.76-88.70) for experienced endoscopists

*CADx,* Computer-assisted diagnosis; *NPV,* negative predictive value; *DRSP,* diminutive rectosigmoid polyp; *CI*, confidence interval.

A key limitation to note with current CADx systems is the lack of user trust. Few of the existing systems provide "explainability" for their decisions, making it less likely that physicians will follow AI recommendations when it goes against a physician decision. It is also unclear if achieving a PIVI threshold of 90% is enough to be accepted in widespread clinical practice in high-resource environments.

Based on these studies, the consensus group voted that CADx can be used to characterize polyps in the colon and rectum. However, there is a distinction between polyp characterization and treatment decision. To date, data are insufficient to support the use of CADx in deciding whether polypectomy should be done at this stage.

Statement 5. CADx currently lacks sufficient accuracy to differentiate SSLs from hyperplastic lesions in the colon.

Level of agreement: A = 74.1%, B = 25.9%, C = 0%, D = 0%, E = 0%

Quality of evidence: III

Classification of recommendation: C

Differentiating SSLs from hyperplastic polyps, both of which have similar endoscopic appearance, is crucial because SSLs have malignant potential and hyperplastic polyps are benign. Polyp management and surveillance recommendations for these 2 types of lesions should differ based on the histology of these polyps.

To date, there is no clinical study designed to evaluate the ability of CADx to differentiate SSLs from hyperplastic lesions during colonoscopy. In a study primarily targeted to compare the accuracy of CADx for polyps, the sensitivity for SSLs was only 17.1% (95% CI, 5.6-28.6).^27^ This is far from satisfactory in characterizing this important lesion with substantial malignant potential. There is also a discrepancy in the classification of SSLs in clinical studies on CADx systems. Some studies considered SSLs as non-neoplastic lesions, whereas others interpreted the results with SSLs regarded as neoplastic lesions. In a few clinical validation studies, SSLs were excluded from the final analysis.^24^^,^^26^ The numbers of SSLs in each of the studies were too small and not powered sufficiently to evaluate the accuracy of CADx in differentiating SSLs from hyperplastic lesions. The consensus group asserted that CADx lacks sufficient accuracy to differentiate SSLs from hyperplastic lesions in the colon. Given that SSLs are increasingly recognized as precursor lesions for the development of CRC, the group strongly supports technologic development for AI-assisted colonoscopy in the detection and characterization of SSLs in the future.

### Computer-assisted quality assurance

Statement 6. CAQ may be used for real-time monitoring of quality assurance during colonoscopy (withdrawal speed, quality of bowel preparation, and mucosal fold examination).

Level of agreement: A = 51.9%, B = 40.7%, C = 3.7%, D = 3.7%, E = 0%

Quality of evidence: I

Classification of recommendation: B

Endoscopy and gastroenterology societies around the world have long emphasized the importance of quality control metrics in colonoscopy, focusing on withdrawal time, bowel preparation quality, cecal intubation rate, and ADR. CAQ for colonoscopy emerges as a transformative approach for real-time quality assurance monitoring during colonoscopy procedures. Evidence demonstrated that CAQ, built on convolutional neural networks, can accurately evaluate bowel preparation with a remarkable 93.33% accuracy, surpassing the performance of many endoscopists in the study.^34^ Real-time enhancements to CAQ modules, including withdrawal time measurement and supervised withdrawal stability, further contribute to its potential to improve the efficiency of colonoscopy examinations.^35^ In a 4-arm, parallel, RCT comprising 1076 cases, the combination of CAQ and CADe outperformed CADe alone, achieving a significantly improved ADR of 30.6% compared with 21.27%.^36^

As CAQ technology evolves, it introduces new quality control measures such as fold examination quality. A recent study showcased significant improvements in ADR (29% [interquartile range, 27-30]) from low ADR (<25%) after using AI-assisted feedback on fold examination quality.^37^ The evolving technology of CAQ holds promise for real-time quality monitoring during colonoscopy, demonstrating its potential to enhance polyp detection, improve endoscopist performance, and facilitate the training of junior endoscopists.

Statement 7. CAQ reporting for colonoscopy should be considered when available to standardize reporting quality.

Level of agreement: A = 48.1%, B = 48.1%, C = 3.7%, D = 0%, E = 0%

Quality of evidence: II-3

Classification of recommendation: C

In addition to quality control of colonoscopy procedures, there is also significant variation in the quality of reporting colonoscopy across diverse medical practices. Implementing structured standard reporting indicators, facilitated by CAQ, provides a valuable solution to identify quality improvement areas within these reports. Integration of CAQ enhances the reporting of new quality control indices. One notable advancement facilitated by CAQ is the introduction of a quantitative bowel preparation score in the report,^38^ replacing the traditional ordinal bowel preparation score (poor, moderate, good). This structured reporting of machine-based assessment in bowel preparation at each segment of the colon allows for a more objective assessment and refined threshold settings.

Standardized reporting through CAQ also enables automated surveillance advice after the colonoscopy.^39^ Studies have suggested that automated surveillance advice, based on updated national and international guidelines for postcolonoscopy surveillance, may be more compliant with these recommendations than advice provided by physicians.^40^ The adoption of CAQ reporting for colonoscopy is recommended when available, because it minimizes missing data and facilitates more accurate and standardized reporting, potentially improving the quality of surveillance advice postcolonoscopy.

### Implementation and training

Statement 8. For successful adoption of AI-assisted colonoscopy in CRC screening, engagement of endoscopists and endoscopy assistants is needed to gain trust.

Level of agreement: A = 74.1%, B = 18.5%, C = 3.7%, D = 3.7%, E = 0%

Quality of evidence: III

Classification of recommendation: C

Knowledge and perception of AI-assisted colonoscopy play a major role in behaviors of endoscopists when deciding whether to adopt these new technologies. In a study from Singapore,^41^ 31.3% of endoscopists disagreed or were ambivalent about using CADe for colonoscopy. There are also concerns on the potential loss of personal contact between doctors and patients. Furthermore, nurses are important stakeholders who provide valuable feedback on implementing new technologies in endoscopy. Studies have shown the benefits of soliciting input from nurses to ensure smooth adoption of innovations and optimize workflows.^42^

There are potential negative impacts of AI endoscopy when it is routinely used in screening and diagnostic procedures. Over-reliance of AI may lead to deskilling of endoscopists, reduction of provider and recipient autonomy in clinical decisions, and potential danger of breaching data privacy of patients. These ethical and professional concerns should be understood by the users of AI-assisted colonoscopy. Understanding the pros and cons of using AI would enhance the trust and acceptance of the technology, whereas actively engaging endoscopy staff in the implementation process would facilitate adoption of AI-assisted colonoscopy.

Statement 9. Knowledge and use of AI-assisted colonoscopy should be included in the training of colonoscopy.

Level of agreement: A = 59.3%, B = 33.3%, C = 7.4%, D = 0%, E = 0%

Quality of evidence: II-3

Classification of recommendation: C

Recent studies have demonstrated the potential benefits of CADe in improving ADR and endoscopic skills, especially for less-experienced trainees.^16^^,^^43^, ^44^, ^45^ An RCT evaluating a real-time CADe system among endoscopists-in-training found that CADe was associated with increased overall ADR compared with standard colonoscopy, particularly for small- to medium-sized adenomas.^16^ A comparative study showed that CADe helped increase sensitivity for detecting polyps and adenomas among early-career endoscopists.^43^ It has been discussed how CADe could act as an educational aid to help steer trainees' visual focus to lesions that could otherwise be missed, although dependency and cognitive overload are potential risks.^44^ AI systems can aid in identifying lesions, potentially making endoscopy training a smoother journey for beginners.^46^ However, there is also a possibility of deskilling, where trainees become overly reliant on technology, impairing development of essential diagnostic skills. A recent study with visual gaze tracking highlighted that because CADe detects polyps faster than humans, its use increased human misinterpretation of normal mucosa and reduced eye travel distance.^47^ Furthermore, CADe can lead to sensory and cognitive overload, when trainees are overwhelmed by excessive alerts of false-positive signals.^48^ A staged approach to integrating CADe technology has been proposed, starting with enhancing trainee literacy, then introducing applications for feedback and metrics tracking, and finally exposing trainees to more advanced diagnostic features.^45^ Overall, the inclusion of AI-assisted colonoscopy in the training curriculum for colonoscopy is recommended.

Statement 10. Patient should be informed when AI-assisted colonoscopy is used in CRC screening.

Level of agreement: A = 48.1%, B = 33.3%, C = 14.8%, D = 3.7%, E = 0%

Quality of evidence: III

Classification of recommendation: C

Although no studies to date have directly looked at informed consent for AI-assisted colonoscopy, a survey conducted in 2021 assessed perspectives on AI use among capsule endoscopy readers across Europe.^49^ The survey found that 51% of respondents believed patients should be made aware about the use of AI in reading their capsule endoscopy examinations and 53% believed patients should not have to expressly consent to the use of AI in capsule endoscopy. Specific information on CADe colonoscopy could include the purpose of AI (how AI assists in detecting polyps or abnormalities), its benefits and limitations, role of AI and clinician (whether AI supports or takes a role in judgement and decision), data privacy measures (data usage and further AI training), and considerations to secure patient autonomy.^50^^,^^51^

## Conclusions

AI-assisted colonoscopy has its value in improving diagnosis and characterization of polyps in the colon and rectum. Future studies should aim to produce evidence for reducing advanced adenoma and CRC as well as its cost-effectiveness. Engagement and training of endoscopists to use AI is also important in successful implementation of this technology. These guidelines would provide an overall set of directions for the use of AI-assisted colonoscopy in CRC screening and surveillance (Table 4). This consensus group noted that more validation studies need to be done while AI technologies continue to improve. Variations in patient and doctor trust, acceptance, healthcare systems, and cost-effectiveness in different jurisdictions should be respected in implementation of this technology.

**Table 4:** Summary of quality of evidence and classification of recommendation of the consensus statements

Statements	Quality of evidence	Classification of recommendation
Statement 1. CADe has been shown to improve adenoma detection.	I	A
Statement 2. CADe should be used if available as a preferred choice over conventional colonoscopy for CRC screening.	II-2	C
Statement 3. In using CADe for CRC screening, adenoma per colonoscopy should be considered as an additional indicator of quality.	II-1	C
Statement 4. CADx can be used for characterization of polyps but is not yet ready to determine the necessity of polypectomy.	II-2	B
Statement 5. CADx currently lacks sufficient accuracy to differentiate sessile serrated lesions from hyperplastic lesions in the colon.	III	C
Statement 6. CAQ may be used for real-time monitoring of quality assurance during colonoscopy (withdrawal speed, quality of bowel preparation, and mucosal fold examination).	I	B
Statement 7. CAQ reporting for colonoscopy should be considered when available to standardize reporting quality.	II-3	C
Statement 8. For successful adoption of AI-assisted colonoscopy in CRC screening, engagement of endoscopists and endoscopy assistants is needed to gain trust.	III	C
Statement 9. Knowledge and use of AI-assisted colonoscopy should be included in the training of colonoscopy.	II-3	C
Statement 10. Patient should be informed when AI-assisted colonoscopy is used in CRC screening.	III	C

*CADe,* Computer-assisted detection; *CADx,* computer-assisted diagnosis; *CAQ,* computer-assisted quality assurance; *CRC,* colorectal cancer; *AI,* artificial intelligence.

## Patient consent

This article does not discuss individual patients, so no consent was needed.

## Disclosure

The following authors disclosed financial relationships: L. H. S. Lau: Research support from Health and Medical Research Fund, General Research Fund, Direct Grant for Research, Olympus Co Ltd, Pfizer Inc, AbbVie Inc, and GenieBiome Ltd; speaker for Olympus Co Ltd, Boston Scientific, and GenieBiome Ltd. J.-S. Byeon: Speaker for Olympus Korea Co Ltd; medical advisor to Ainex Corporation. P. Sharma: Research support from Erbe, 10.13039/501100002424Fujifilm, NEC, and Sebela; consultant for Olympus Corporation, Boston Scientific, Salix Pharmaceuticals, Cipla, Medtronic, Takeda, Samsung Bioepis, and CDx. All other authors disclosed no financial relationships.
